# Serum fetuin-A is associated with the components of MIAC(malnutrition, inflammation, atherosclerosis, calcification) syndrome in different stages of chronic kidney disease

**DOI:** 10.3906/sag-1809-43

**Published:** 2019-02-11

**Authors:** Rüya MUTLUAY, Ceyla KONCA DEĞERTEKİN, Emel IŞIKTAŞ SAYILAR, Ülver DERİCİ, Serap GÜLTEKİN, Sevim GÖNEN, Selim Turgay ARINSOY, Mahmut Şükrü SİNDEL

**Affiliations:** 1 Department of Nephrology, Yunus Emre State Hospital, Eskişehir Turkey; 2 Department of Endocrinology, Faculty of Medicine, Gazi University, Ankara Turkey; 3 Department of Nephrology, Edirne Sultan I. Murat State Hospital, Edirne Turkey; 4 Department of Nephrology, Faculty of Medicine, Gazi University, Ankara Turkey; 5 Department of Radiology, Faculty of Medicine, Gazi University, Ankara Turkey

**Keywords:** Atherosclerosis, calcification, chronic renal failure, fetuin-A, inflammation, malnutrition

## Abstract

**Background/aim:**

Fetuin-A, a circulating inhibitor of calcification, is a marker of inflammatory-nutritional state. We evaluated the association between serum fetuin-A levels and vascular calcification, intima-media thickness, and nutritional and inflammatory markers in different stages of chronic kidney disease (CKD).

**Materials and methods:**

CKD patients were sampled for calcium-phosphate parameters and nutritional and inflammatory markers [highly sensitive C-reactive protein (hs-CRP)], and serum fetuin-A levels. Intima-media thicknesses of the common carotid arteries (CIMT) were measured. Peripheral artery calcification scores were obtained.

**Results:**

A total of 238 patients were included in the study. Fetuin-A levels in patients with end-stage renal disease were significantly lower than those in patients with stage-3 and stage-4 CKD (stage-5 vs. stage-4, P < 0.001; stage-5 vs. stage-3, P < 0.001). Fetuin-A was negatively correlated with creatinine (P < 0.001), Ca × P product (P < 0.001), hs-CRP (P = 0.01), vascular calcification score (P < 0.001), and CIMT (P < 0.001), and positively correlated with BMI (P < 0.001, r = 0.30) and serum albumin (P < 0.001).

**Conclusion:**

Lower levels of fetuin-A were associated with higher vascular calcification scores, CIMT, hs-CRP levels, and lower BMI and albumin. Fetuin-A deficiency may be a key element for MIAC syndrome.

## 1. Introduction

Progressive vascular calcification is a major cause of cardiovascular morbidity and mortality in patients with end-stage renal disease (ESRD) [1]. The development of vascular calcification is a problem even for patients with mild to moderate kidney dysfunction [2]. It is a continuum, probably starting at the very early stages of decreasing renal reserve. The presence of abnormal mineral metabolism and other inducers of vascular calcification, as well as decreased expression of calcification inhibitors such as matrix Gla protein, fetuin-A, osteoprotegerin, and osteopontin facilitate the development and progression of vascular calcification [3].

Fetuin-A is a serum glycoprotein derived predominantly from liver. It prevents precipitation of calcium and phosphate and acts as a potent systemic calcification inhibitor [4]. Several studies reported an association between low levels of serum fetuin-A and vascular/valvular calcifications, poor clinical outcome, and increased mortality in patients with ESRD [5–8]. It was shown to be an independent risk factor for progressive arterial stiffness [9] and endothelial dysfunction [10]. Fetuin-A is also known as a negative acute phase reactant; its concentration falls during inflammation [11] and inflammatory processes are known to play a role in the formation of atherosclerosis [12]. Malnutrition-inflammation-atherosclerosis-calcification (MIAC) syndrome is the current definition of this interplay between inflammation and atherosclerosis in uremic patients. Fetuin-A might have a close relationship with this entity since its levels fall across the groups of patients with increasing components of the MIAC syndrome [8]. 

Based on the present data, the precise role of fetuin-A in uremic patients has not been strongly established yet and very few studies have investigated the role of fetuin-A in patients with stage-3 and stage-4 chronic kidney disease (CKD). In this study, we aimed to determine (i) whether a correlation exists between fetuin-A and hs-CRP levels, nutritional parameters, carotid intima-media thickness (CIMT) or vascular calcification scores (VCS) in stage-3, stage-4, and ESRD patients, (ii) whether fetuin-A, hs-CRP levels, nutritional parameters, CIMT, and VCS are different in patients with stage-3 and stage-4 CKD compared to patients with ESRD.

## 2. Materials and methods

### 2.1. Patient population and study design 

A total of 238 patients with varying degrees of renal insufficiency were included in the study. They were grouped based on estimated glomerular filtration rate (GFR), as calculated by the simplified MDRD Study prediction equation [13]. Patients with GFR values of 30–59 mL/min were grouped as stage-3 CKD, 15–29 mL/min as stage-4 CKD, and <15 mL/min as stage-5 CKD according to National Kidney Foundation. One hundred and seven of the patients had stage-3 and stage-4 CKD (stage-3 CKD, n = 62; stage-4 CKD, n = 45) and none of them was undergoing renal replacement therapy. One hundred and thirty-one of the patients had ESRD (stage-5 CKD) and all were undergoing renal replacement therapy. The ESRD group was composed of 68 hemodialysis (HD) patients undergoing standard bicarbonate dialysis routinely three times a week and 63 continuous ambulatory peritoneal dialysis (CAPD) patients. The study was carried out with chronic kidney disease patients (dialysis and predialysis) who were followed up in the nephrology outpatient clinic of the Faculty of Medicine, Gazi University in an ambulatory setting. The patient recruitment lasted for 2 years. Participants younger than 18 years or older than 70 years old, those with underlying malignancy, chronic liver disease, autoimmune disease, current or recent (<1 month) active infection, a catheter or graft as vascular access (for HD group) and those with a history or symptoms of cardiovascular disease and cardiovascular instability in the past (myocardial infarction, congestive heart failure, arrhythmia, peripheral vascular disease, transient ischemic attacks, or cerebrovascular accidents) were not included in the study. The demographic and clinical data were collected from patient charts. None of the patients were receiving statins or renin-angiotensin system blocking agents for at least the last 4 weeks before enrolling in the study. None of the patients in the stage-3 and stage-4 CKD groups was on erythropoietin, active vitamin D, or phosphorus binder (calcium-based or sevalemer) therapy. In the HD group, 14 (21%) patients were receiving erythropoietin alfa, 12 (18%) erythropoietin beta, and 8 (12%) darbepoetin alfa while in the CAPD group, 18 (29%) were using darbepoetin alfa and 10 (16%) erythropoietin beta. Ten (15%) of the patients were using sevalemer and 24 (35%) calcium acetate in the HD group; in the CAPD group, the numbers were 3 (5%) and 23 (37%), respectively. Twenty-nine percent of the HD patients were receiving active vitamin D while the rate was 26% in the CAPD group. Table 1 summarizes the demographic and etiologic distribution of patients.

**Table 1:** Characteristics of the study group.

	Stage-3 (n = 62)	Stage-4 (n = 45)	Stage-5 (n = 131)	P
Male/female (n)	39/23	22/23	79/52	0.30
Etiology				
Hypertension	24	22	42	
Diabetes mellitus	22	12	24	
Glomerulonephrotis	5	2	20	
Nephrolithiasis	5	3	7	
Amiloidosis	1	2	5	
Polycystic kidney disease	1	1	4	
Pyelonephritis	1	-	5	
Unknown	3	3	24	
Age (years)	54.17 ± 6.92	55.02 ± 11.36	51.74 ± 12.96	0.16
BMI (kg/m2)	28.58 ± 5.07	27.13 ± 4.45	24.49 ± 5.28	<0.001
Albumin (g/dL)	4.45 ± 0.49	4.16 ± 0.43	4.05 ± 0.43	<0.001
Hemoglobin (g/dL)	12.66 ± 1.73	11.48 ± 1.25	10.82 ± 1.56	<0.001
Creatinine (mg/dL)	1.87 ± 0.44	2.89 ± 1.01	8.93 ± 2.81	<0.001
Calcium (mg/dL)	9.09 ± 0.51	8.8 ± 0.62	8.87 ± 0.75	0.05
Phosphorus (mg/dL)	3.64 ± 0.54	4.08 ± 0.80	5.19 ± 1.39	<0.001
Ca × P (mg2/dL2)	33.00 ± 4.55	35.75 ± 6.83	46.15 ± 13.35	<0.001
iPTH (ng/mL)	102.17 ± 60.61	175.97 ± 110.54	442.93 ± 419.21	<0.001
VCS	0.43 ± 0.96	0.51 ± 0.86	1.9 ± 2.08	<0.001
Hs-CRP (mg/dL)	0.54 ± 0.65	0.49 ± 0.36	0.85 ± 1.04	0.01
Total chol. (mg/dL)	191.54 ± 38.27	185.75 ± 47.05	179.62 ± 39.77	0.16
Triglyceride (mg/dL)	167.33 ± 81.31	162.26 ± 67.06	186.47 ± 113.67	0.24
m-CIMT (mm)	0.61 ± 0.50	0.69 ± 0.43	0.95 ± 0.31	<0.001
Fetuin-A (ng/mL)	66.09 ± 9.68	66.81 ± 13.17	10.31 ± 1.09	<0.001

Data presented as mean ± SD; BMI: body mass index, Ca × P: calcium phosphorus product, iPTH: intact Parathormone, VCS: vascular calcification score, Hs-CRP: highly sensitive CRP, Total chol.: total cholesterol, m-CIMT: mean carotid intima-media thickness.

The study was approved by the local ethical committee and written informed consent was obtained from all patients prior to enrolling in the study. The study was conducted according to the declaration of Helsinki.

### 2.2. Measurement of biochemical parameters, hs-CRP, and fetuin-A 

Venous blood from patients were collected for measurement of biochemical parameters, blood count, hs-CRP, and fetuin-A. Blood was drawn from a peripheral vein in the morning hours, after an overnight fast of 8 h, before the dialysis session in the HD group and before the first peritoneal dialysis exchange of the day in the CAPD group. The serum and EDTA plasma aliquots were stored at −70 °C until analysis. Routine biochemical variables and blood counts were measured by standardized methods using autoanalyzers. The biochemical variables were creatinine, calcium, phosphorus, albumin, total cholesterol, HDL cholesterol, triglycerides. LDL cholesterol levels were calculated according to the formula described by Friedewalds et al. [14]. Serum intact parathyroid hormone (iPTH) levels were measured with the immunoradiometric assay (IRMA) with a commercially available kit. The highly sensitive C-reactive protein (hs-CRP) levels were measured with the nephelometric method. Serum fetuin-A was analyzed using a human fetuin-A enzyme linked immunosorbent assay (ELISA) kit (BioVendor GmbH, Heidelberg, Germany) and the results were expressed as ng/mL. 

### 2.3. Measurement of carotid intima media thickness 

The carotid arteries were evaluated in all patients and with a high-resolution real-time B-mode ultrasonography (ATL HDI 5000, Philips, Bothell, WA, USA) using a 13.5 MHz linear array transducer. The ultrasonography examination was performed by an experienced radiologist, kept unaware of other data. Subjects were examined in the supine position. Common carotid arteries (CCA) were examined bilaterally in all patients, scanned in multiple longitudinal and transverse planes to determine the best view perpendicular to the vessel wall for measuring IMT. The measurements were obtained from a region 10 mm proximal to the carotid bifurcation for CCA. The CIMT was measured in the far wall of the vessel as the distance from the leading edge of the lumen-intima interface to the leading edge of the media-adventitia interface. The averages of the four measurements for each side were determined. The arithmetic mean value of right and left measurements was taken as the mean CIMT (m-CIMT) [15].

### 2.4. Scoring vascular calcification 

Vascular calcification was scored using plain radiographic films of pelvis and hands, as proposed by Adragao et al. The pelvis films were divided into four sections and films of both hands, each into two sections, by imaginary lines (a horizontal line over the upper limit of both femoral heads and a median vertical line over the vertebral column for pelvis, a horizontal line over the upper limit of the metacarpal bones for hands). In each section, the presence of linear calcification was scored as 1 and the VCS was determined by counting up the scores in all sections, ranging from 0 to 8. Linear calcifications along the iliac and femoral arteries in pelvis film and radial and digital arteries in hand films were considered when scoring. Patchy or extravascular calcifications were not counted [16].

### 2.5. Statistical analysis 

The results were expressed as mean + SD. In all cases, comparisons were two-tailed, and a P-value of <0.05 was considered statistically significant. The differences among the three groups for parametric variables [age, BMI, m-CIMT, fetuin-A, hemoglobin, creatinine, calcium (Ca), phosphorus (P), calcium–phosphorus product (Ca × P), iPTH, total cholesterol, HDL, LDL, albumin, hs-CRP, VCS, triglycerides) were assessed using the one-way ANOVA and Tukey HSD test and the nonparametric variables were assessed using the chi-square test. For one-to-one group comparison of parametric variables, the t-test was used. The patients were stratified into tertiles according to serum fetuin-A concentrations: fetuin-A 0.30–10.59 ng/mL (first tertile), 10.60–61.99 ng/mL (second tertile), and 62.00–79.00 ng/mL (third tertile). Correlations between the variables were analyzed with Pearson’s correlation analysis. Backward multiple linear regression analyses were performed to test the associations between several possible associated factors and fetuin-A, VCS, and CIMT separately. Variables with P < 0.05 on univariate analyses (BMI, hemoglobin, albumin, creatinine, Ca × P, iPTH, hs-CRP, VCS, and m-CIMT for fetuin-A; creatinine, Ca × P, iPTH, hs-CRP, BMI, hemoglobin, albumin, fetuin-A, and CIMT for VCS; age, VCS, creatinine, hs-CRP, BMI, hemoglobin, albumin, and fetuin-A for CIMT) were entered into the multiple regression models. Given the interaction between serum P, Ca, and Ca × P, we have excluded serum P/Ca from the regression models despite its significant associations on univariate analysis to avoid collinearity. Age was included in the model for VCS and LDL cholesterol and Ca × P for CIMT despite nonsignificance on univariate analysis. SPSS version 10.0 (SPSS, Inc., Chicago, IL, USA) was used to perform all statistical calculations.

## 3. Results

### 3.1. Baseline characteristics of the groups

A total of 238 patients with varying degrees of renal insufficiency were included in the study. There were 131 stage-5 (ESRD), 45 stage-4, and 62 stage-3 CKD patients. The age and sex distributions were similar among the patient groups. Hypertension and diabetes were the most frequent causes of kidney disease for all stages, followed by glomerulonephritis, nephrolithiasis, amyloidosis, polycystic kidney disease, and pyelonephritis. The etiology of the kidney disease was unknown for 3 patients in stage 3, 3 patients in stage 4, and 24 patients in stage 5. Table 1 summarizes the baseline characteristics of the participants.

Patients’ BMI showed significant differences among the groups. Stage-3 and stage-4 patients had significantly higher BMI (stage-3 vs. stage-5, P < 0.001; stage-4 vs. stage-5, P = 0.003) compared to the ESRD patients. Both serum albumin and hemoglobin levels decreased as the stage of the kidney disease progressed. Stage-4 and stage-5 patients had significantly lower serum albumin levels than stage-3 patients (stage-3 vs. stage-4, P = 0.002; stage-4 vs. stage-5, P = 0.18; stage-3 vs. stage-5, P < 0.001). Hemoglobin levels were significantly different among groups (stage-3 vs. stage-4, P < 0.001; stage-4 vs. stage-5, P = 0.01; stage-3 vs. stage-5, P < 0.001). Baseline laboratory tests are summarized in Table 1. 

When the HD and CAPD patients were compared, they had similar duration of dialysis, hemoglobin and albumin levels but the HD patients had a lower BMI than the CAPD patients (Table 2).

**Table 2:** Characteristics of the HD and CAPD patients.

	HD (n = 68)	CAPD (n = 63)	P
Duration of dialysis (months)	70.34 ± 31.13	68.36 ± 32.14	0.31
BMI (kg/m2)	23.34 ± 4.53	25.63 ± 5.58	0.02
Hemoglobin (g/dL)	10.69 ± 1.35	11 ± 1.82	0.25
Albumin (g/dL)	4.08 ± 0.49	4.01 ± 0.36	0.39
Ca × P (mg2/dL2)	46.34 ± 12.11	45.82 ± 15.08	0.81
iPTH (ng/mL)	420.48 ± 419	477.61 ± 425.12	0.41
Hs-CRP (mg/dL)	0.82 ± 1.21	0.86 ± 0.76	0.08
VCS	2.18 ± 2.37	1.64 ± 1.21	0.13
m-CIMT (mm)	1.01 ± 0.32	0.95 ± 0.36	0.06
Fetuin-A (ng/mL)	10.16 ± 1.40	10.49 ± 0.47	0.08

Data presented as mean ± SD; BMI: body mass index, Ca × P: calcium–phosphorus product, iPTH: intact Parathormone, VCS: vascular calcification score, Hs-CRP: highly sensitive CRP, m-CIMT: mean carotid intima-media thickness.

### 3.2. Fetuin-A and other study parameters

There was a significant increase in Ca × P and iPTH levels as the stage of kidney disease progressed. Stage-4 patients had higher Ca × P and iPTH levels compared to stage-3 (for Ca × P, P = 0.01; for iPTH, P < 0.001) and stage-5 compared to stage-4 (for Ca × P, P < 0.001; for iPTH, P < 0.001). VCS had a similar trend of increase as the Ca × P and iPTH levels with the highest scores in the ESRD patients. The difference was significant between stage-5 and stage-4 (P = 0.009) or stage-3 (P < 0.001) patients but not between stage-3 and stage-4 (P = 0.28) patients. Hs-CRP levels of the stage-3 and 4-patients (P = 0.67) were similar but they were significantly higher in the ESRD group compared to both stage-3 (P = 0.03) and stage-4 (P = 0.02) patients. The m-CIMT for stage-3, -4, and -5 patients were 0.61 ± 0.50, 0.69 ± 0.43, and 0.95 ± 0.31 mm, respectively. The difference in the m-CIMT was statistically significant when the ESRD and stage-3 (P < 0.001) or stage-4 (P < 0.001) CKD patients were compared. Although the score was higher in patients with stage-4 CKD compared to stage-3, the difference was not statistically significant (P = 0.38). The mean serum fetuin-A concentrations for stage-3, -4, and -5 CKD were 66.09 ± 9.68, 66.81 ± 13.17, and 10.31 ± 1.09, respectively. Fetuin-A in patients with ESRD was significantly lower than in patients with CKD (stage-5 vs. stage-4, P < 0.001; stage-5 vs. stage-3, P < 0.001) but the difference was not significant between stage-3 and stage-4 patients (P = 0.74). When the stage-5 patients were analyzed according to their mode of renal replacement separately, there were no differences between Ca × P, iPTH, VCS, hs-CRP, m-CIMT, and serum fetuin-A levels (Table 2). 

The mean HbA1C levels of the 58 diabetic patients included in the study was 7.21 ± 1.76. There was no statistically significant relationship between HbA1C and fetuin-A levels. The age and sex distributions were similar among fetuin-A tertiles. Patients in the highest tertile had higher BMI, albumin, and hemoglobin levels and lower creatinine, P, Ca × P, iPTH, VCS, and m-CIMT than those in the middle and lowest tertiles (Table 3).

**Table 3:** Basic characteristics of patients in tertiles of fetuin-A concentration.

	All patients (n = 238)	Fetuin-A tertile (ng/mL)			P
		0.30–10.59 (n = 79)	10.60–61.99 (n = 81)	62.00–79.00 (n = 78)	
Age (years)	53.00 ± 11.43	52.94 ± 13.14	51.94 ± 11.72	54.15 ± 9.04	0.475
Sex (woman %)	41	38	38.3	47.4	0.393
BMI (kg/m2)	26.06 ± 5.37	24.60 ± 5.09	25.78 ± 6.01	27.83 ± 4.43	0.001
Albumin (g/dL)	4.18 ± 0.48	4.11 ± 0.41	4.12 ± 0.54	4.32 ± 0.46	0.009
Hemoglobin (g/dL)	11.43 ± 1.74	10.79 ± 1.63	11.40 ± 1.83	12.10 ± 1.50	<0.001
Creatinine (mg/dL)	5.95 ± 3.95	9.19 ± 2.86	6.29 ± 3.76	2.33 ± 0.95	<0.001
Calcium (mg/dL)	8.92 ± 0.68	8.93 ± 0.81	8.82 ± 0.67	9.00 ± 0.54	0.270
Phosphorus (mg/dL)	4.58 ± 1.32	5.18 ± 1.36	4.67 ± 1.40	3.87 ± 0.76	<0.001
Ca × P (mg2/dL2)	40.76 ± 12.18	46.28 ± 13.03	41.32 ± 13.08	34.60 ± 6.09	<0.001
iPTH (ng/mL)	303.69 ± 352.21	473.06 ± 433.17	297.49 ± 347.00	138.59 ± 97.78	<0.001
VCS	1.27 ± 1.82	1.81 ± 2.16	1.56 ± 1.86	0.42 ± 0.85	<0.001
Hs-CRP (mg/dL)	0.70 ± 0.87	0.80 ± 0.92	0.75 ± 1.01	0.55 ± 0.60	0.173
Total chol. (mg/dL)	183.89 ± 41.01	180.09 ± 39.24	177.63 ± 44.68	194.24 ± 37.12	0.022
Triglyceride (mg/dL)	176.91 ± 98.69	190.72 ± 132.69	168.31 ± 74.09	171.86 ± 78.36	0.308
m-CIMT (mm)	0.82 ± 0.42	0.97 ± 0.34	0.85 ± 0.37	0.62 ± 0.47	<0.001

Data presented as mean ± SD; BMI: body mass index, Cax × P: calcium–phosphorus product, iPTH: intact Parathormone, VCS: vascular calcification score, Hs-CRP: highly sensitive CRP, Total chol.: total cholesterol, m-CIMT: mean carotid intima-media thickness.

### 3.3. Results of the multiple linear regression analysis

There was a positive correlation between fetuin-A levels and BMI (P < 0.001, r = 0.30), hemoglobin (P < 0.001, r = 0.37), and albumin (P < 0.001, r=0.26) levels; total cholesterol (P = 0.05, r = 0.12) was not correlated but showed a trend (Table 4). Fetuin-A was negatively correlated with creatinine (P < 0.001, r = −0.80), P (P < 0.001, r = −0.48), Ca × P (P < 0.001, r = −0.46), iPTH (P < 0.001, r = −0.41), hs-CRP (P = 0.01, r = −0.16), VCS (P < 0.001, r = −0.40), and m-CIMT (P < 0.001, r = −0.37). In the stage-5 group undergoing dialysis, fetuin-A levels were not correlated with the duration of dialysis (P = 0.17, r = −0.12). Fetuin-A levels were independently associated with m-CIMT, VCS, creatinine and albumin levels on multiple regression analysis (Table 5). In univariate analysis, m-CIMT was positively correlated with age, VCS, creatinine, P, and hs-CRP and negatively correlated with BMI, hemoglobin, Ca, albumin, and fetuin-A (Table 4). When multiple regression analysis was performed, we found that fetuin-A as well as VCS, age, and hemoglobin were independent predictors of m-CIMT (Table 5). VCS was positively correlated with duration of dialysis, creatinine, phosphorus, Ca × P, iPTH, and hs-CRP, and negatively correlated with BMI, hemoglobin, albumin, and fetuin-A in univariate analysis (Table 4). We found that VCS was independently associated with fetuin-A, albumin, and m-CIMT when multiple regression analysis was performed (Table 5).

**Table 4:** Correlations between fetuin-A, m-CIMT, and study parameters.

Variables	r	P
	Fetuin-A	
BMI	0.30	<0.001
Hemoglobin	0.37	<0.001
Albumin	0.26	<0.001
Total cholesterol	0.12	0.05
Creatinine	–0.80	<0.001
Phosphorus	–0.48	<0.001
Ca × P	–0.46	<0.001
iPTH	–0.41	<0.001
hs-CRP	–0.16	0.01
VCS	–0.40	<0.001
m-CIMT	–0.37	<0.001
	m-CIMT	
Age	0.14	0.02
VCS	0.34	<0.001
Creatinine	0.29	<0.001
Phosphorus	0.15	0.02
hs-CRP	0.14	0.02
BMI	–0.13	0.04
Hemoglobin	–0.30	<0.001
Calcium	–0.13	0.03
Albumin	–0.13	0.03
Fetuin-A	–0.37	<0.001
	VCS	
Duration of dialysis	0.25	0.003
Creatinine	0.28	<0.001
Phosphorus	0.16	0.009
Ca × P	0.16	0.01
iPTH	0.19	0.002
hs-CRP	0.15	0.01
BMI	–0.19	0.003
Hemoglobin	–0.19	0.003
Albumin	–0.26	<0.001
Fetuin-A	–0.40	<0.001

BMI: body mass index, Ca × P: calcium–phosphorus product, iPTH: intact Parathormone, hs-CRP: highly sensitive CRP, VCS: vascular calcification score, m-CIMT: mean carotid intimamedia thickness.

**Table 5:** Multiple regression analyses of factors affecting VCS, m-CIMT, and fetuin-A levels.

Dependent	Independent	Beta	P
VCS	Fetuin-A	–0.283	<0.001
	Albumin	–0.155	0.01
	m-CIMT	0.216	0.001
Removed variables	Ca × P, age, creatinine, BMI,hs-CRP, hemoglobin, iPTH		
m-CIMT	Fetuin-A	–0.236	<0.001
	VCS	0.203	0.001
	Age	0.175	0.003
	Hemoglobin	–0.192	0.002
Removed variables	BMI, hs-CRP, LDL-C,creatinine, Ca × P, albumin		
Fetuin-A	m-CIMT	–0.097	0.013
	VCS	–0.125	0.002
	Creatinine	–0.725	<0.001
	Albumin	0.151	<0.001
Removed variables	Hemoglobin, iPTH, Ca × P,hsCRP, BMI		

m-CIMT: mean carotid intima-media thickness, VCS: vascular calcification score, BMI: body mass index, Ca × P: calcium–phosphorus product, iPTH: intact Parathormone, Hs-CRP: highly sensitive CRP.

## 4. Discussion

In this study, we observed that fetuin-A levels were negatively correlated with serum creatinine and were significantly lower in patients with ESRD compared to patients with CKD. There are conflicting results in the literature on how fetuin-A levels change with renal dysfunction. Data from the Heart and Soul study, recruiting a group of participants with coronary artery disease and variable renal function showed that fetuin-A was not associated with GFR assessed by different methods [17]. Another study reported by Mehrotra et al. emphasizes the effect of ethnicity on serum fetuin-A levels in a cohort of diabetic patients. They showed that Latinos with reduced renal clearance and significant proteinuria had higher serum fetuin-A levels compared to Latinos without nephropathy. Interestingly, serum fetuin-A levels were similar between African–American diabetic nephropathy patients and Latinos without nephropathy [18]. However, more recent studies reported a negative association between fetuin-A and renal function. Caglar et al. demonstrated that in a group of nondiabetic patients with different stages of CKD, fetuin-A levels decreased with a reduction in GFR [10]. Similarly, in a study involving patients with stage-3, -4, and -5 CKD (cohort of both diabetics and nondiabetics with no patients on dialysis), plasma fetuin-A levels progressively decreased with worsening renal function [19]. Mazzafero et al. showed that fetuin-A levels were lower in dialysis patients compared to renal transplantation patients and healthy controls [20]. We believe that different methodologies used to assess renal function, ethnical diversity, presence or absence of diabetes, and the different number of patients included in these studies may explain these controversial results.

Previous studies reported a relation between fetuin-A and the presence of coronary and valvular calcification in ESRD patients [21,22]. Peripheral vascular calcification in relation to fetuin-A has been evaluated much less frequently in uremic patients. In a very recent study, Coen et al. investigated the histology and computed tomography images of the lower epigastric artery in 44 HD patients and suggested that there was a linkage between serum fetuin-A and fibroblast growth factor 23 levels and peripheral arterial calcification. A study conducted by Ulutas et al., which investigated 93 patients with ESRD, no correlation was found between serum fetuin-A and the severity of VCS [23]. The VCS in our patients increased with worsening renal function. The difference was statistically significant between the ESRD and the CKD patients. In our study, we preferred plain X-rays to determine VCS because the other noninvasive complicated techniques, such as electron beam computed tomography and multislice computed tomography are expensive and not widely available for large populations in our country. We found a significant negative correlation between fetuin-A and calcification scores as well as Ca × P and we believe that this may support the discussion that deficient calcification inhibitors may play a key role in unwanted calcification given that the balance of Ca and P metabolism is disturbed. 

We also found that CIMT measurements increased progressively with the progression of renal failure and this increase was negatively correlated with fetuin-A levels. These results are in concordance with a study where 60 pediatric patients with different stages of CKD were investigated and lower fetuin-A levels were associated with higher CIMT values [24]. Although serum fetuin-A levels were similar between the CKD and the ESRD patients in this study, it was the only parameter significantly related to CIMT in all patients. However, these results were not confirmed by Schlieper et al., who found no correlation between fetuin-A levels and CIMT and pulse wave velocity in HD patients [25]. In another study conducted on a population of nondiabetic subjects at increased risk for type-2 diabetes and cardiovascular disease, a positive correlation between CIMT and serum fetuin-A levels were reported; however, GFR distribution of the population was not specified [26].

In our study, the CIMT measurements were also correlated with VCS. There are studies in the literature reporting that coronary and valvular calcifications may be accompanied by intima-media thickening [27,28] but to the best of our knowledge, the association between CIMT and peripheral VCS has not been reported previously. 

We found that lower fetuin-A levels were associated with higher hs-CRP and lower albumin levels. Previous studies had reported negative correlations between fetuin-A levels and inflammatory markers as hs-CRP [29] and proinflammatory cytokines as IL-6 and TNF-α [30] in dialysis patients. Interestingly, Metry et al. reported that low levels of fetuin-A predicted higher mortality only in the presence of elevated CRP on HD patients [7]. Based on these findings, we believe that inflammation appears to contribute to the calcification process. 

BMI and serum albumin levels, as predictors of nutrition, were positively correlated with fetuin-A levels in our study. Similar to our results, in a group of nondiabetic HD patients, higher fetuin-A levels were associated with greater risk for truncal obesity independent of nutritional status and inflammation [31]. In another study on (peritoneal dialysis) PD patients, higher albumin was associated with higher fetuin-A levels [8]. Coen et al*.* reported that HD patients with lowest tertile of fetuin-A had the highest CRP levels and fetuin-A was associated with prealbumin. They concluded that fetuin-A may be linked to atherosclerosis via inflammatory-nutritional pathway as well as through increased vascular calcification [21]. Whether due to or independent of inflammation, malnutrition may be associated with low fetuin-A levels. However, most of the data comes from ESRD patients and the relation of fetuin-A to inflammation and malnutrition in CKD patients seems to be vague.

In our study, we found that fetuin-A levels were individually associated with the components of MIAC syndrome: calcification through VCS, atherosclerosis through CIMT, inflammation through hs-CRP, and malnutrition indirectly through BMI and albumin levels, in CKD patients as well as ESRD patients. A prospective analysis by Wang et al. demonstrated that patients presenting with all components of the MIAC syndrome had the lowest level of fetuin-A and these low levels were associated with all-cause mortality among 238 patients on peritoneal dialysis [8]. We assume that fetuin-A deficiency may be a key element of the MIAC syndrome.

We should consider the limitations of our study, which are the cross-sectional design and single point measurement of markers of interest; thus, future morbidity and mortality could not be predicted. We are following these patients and will hopefully present the follow-up data in the years to come.

In conclusion, lower levels of fetuin-A were associated with higher VCS, higher CIMT, higher hs-CRP levels, lower BMI, and lower levels of albumin in patients not yet on renal replacement therapy as well as patients with ESRD.

## Acknowledgments

This work was funded by a grant from Gazi University, Ankara, Turkey. The study sponsors had no role in study design, data collection, data analysis, data interpretation, and/or writing of the report. The authors declare that they have no competing interests.
